# Plaie transfixiante de l’avant-bras par implantation d’un objet tranchant inhabituel: à propos d’un cas

**DOI:** 10.11604/pamj.2021.39.186.30493

**Published:** 2021-07-08

**Authors:** Laurent Désiré Ndzié Essomba, Hamadassaliha Agaly, Youssouf Diallo, Abdoul Kadri Moussa

**Affiliations:** 1 Service de Chirurgie Orthopédique et Traumatologique, Centre Hospitalier Universitaire Gabriel Touré, Bamako, Mali,; 2 Service de Neurochirurgie, Centre Hospitalier Universitaire Gabriel Touré, Bamako, Mali,; 3 Service de Chirurgie Orthopédique et Traumatologique, Centre Hospitalier Universitaire Gabriel Touré, Université des Sciences, des Techniques et des Technologies de Bamako (USTTB), Bamako, Mali

**Keywords:** Plaie transfixiante, coup de couteau, avant-bras, lésions neurovasculaires, à propos d’un cas, Transfixed wound, knife stroke, forearm, neurovascular lesions, case report

## Abstract

Les plaies traumatiques transfixiantes des membres avec corps étranger resté implanté sont rares. L´atteinte du segment antébrachial peut entrainer des lésions tissulaires nécessitant un traitement chirurgical spécialisé. Peu d´études concernant ces cas ont été rapportées, une ablation chirurgicale du corps étranger est indiquée. Nous rapportons le cas d´un patient de 23 ans, victime de violences physiques, présentant une plaie transfixiante par implantation d´un couteau atypique à l´avant-bras. L´examen clinique et radiographique a diagnostiqué des lésions musculaires et neurovasculaires importantes. L´intervention chirurgicale a permis le retrait du couteau et la réparation des lésions. La prise en charge de tels traumatismes doit être faite par une équipe chirurgicale expérimentée afin d´éviter la survenue de lésions iatrogènes.

## Abstract (en)

Traumatic transfixed wounds of the limbs due to embedded foreign body are rare. Antebrachial segment involvement can lead to tissue damage, requiring specialized surgical treatment. Few studies have been reported, but surgical removal of the foreign body is indicated. We report the case of a 23-year-old patient, victim of physical violence, presenting with a transfixed wound due to the implantation of an atypical knife in the forearm. Clinical and radiographic tests showed significant muscle and neurovascular lesions. Surgical removal of the knife and lesion repair were performed. Surgical treatment of these traumas must be performed by an experienced surgical team to avoid the occurrence of iatrogenic lesions.

## Introduction

Les plaies transfixiantes des membres par armes blanches avec implantation d´objet traumatisant sont rares. L´atteinte du segment antébrachial entraine souvent des lésions multi-tissulaires; osseuses, musculaires et neurovasculaires [1]. Devant une suspicion de blessures complexes avec une arme blanche, la prise en charge est une urgence médicochirurgicale qui doit être faite par une équipe expérimentée. L´objectif est double: retirer l´objet traumatisant sans causer de lésions iatrogènes, traiter de façon optimale les lésions potentiellement graves pouvant compromettre le pronostic vital ou fonctionnel du membre. Nous rapportons le cas d´une plaie transfixiante de l´avant-bras par une arme blanche non habituelle.

## Patient et observation

**Information du patient:** homme de 23 ans, droitier, tabagique, admis au service des urgences 45 minutes après le traumatisme, provenant du centre de santé de sa localité, avec un couteau implanté dans l´avant-bras gauche survenu au cours d´une rixe. Il se plaignait d´une douleur vive de l´avant-bras et d´une impotence fonctionnelle totale du membre traumatisé.

**Figure 1:**
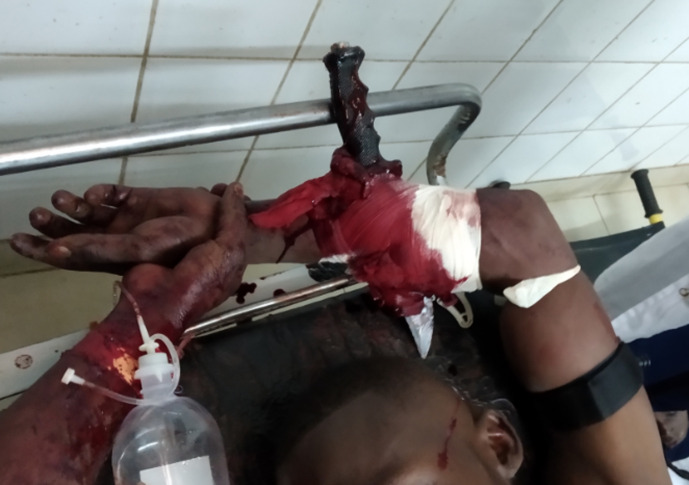
patient à son arrivée au service des urgences avec un couteau transfixiant le 1/3 moyen de l’avant-bras gauche

**Résultats cliniques:** il avait bonne coloration conjonctivale, sa tension artérielle était de 120/70 mmHg. L´examen du membre retrouvait un pansement taché de sang enveloppant un couteau implanté de part en part au 1/3 moyen de l´avant-bras. On notait la présence d´un garrot tourniquet sur son bras (Figure 1).

**Figure 2:**
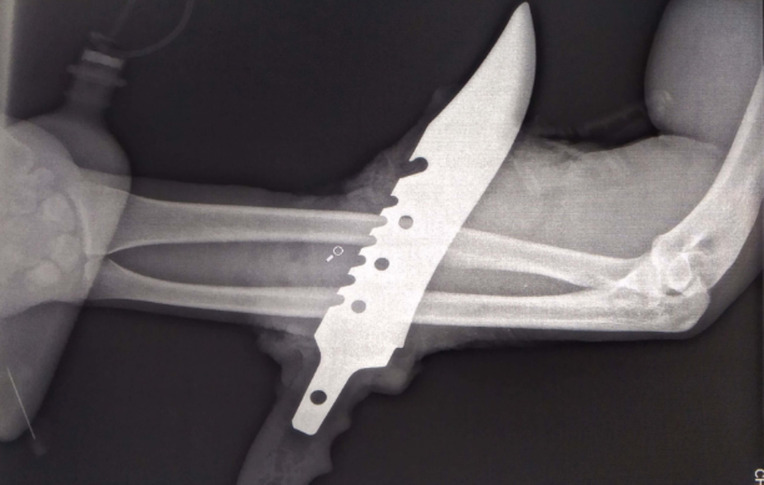
radiographie de l’avant-bras gauche montrant le type de couteau, son siège et sa direction

**Démarche diagnostique:** la levée momentanée du garrot permettait de noter la disparition en distalité du pouls radial avec conservation du pouls ulnaire, un temps de recoloration des doigts de 3 secondes. L´examen neurologique du membre était difficile devant le caractère hyperalgique et agité du patient. On notait une mobilité limitée des doigts. L´analyse radiographique permettait de relever la présence d´un couteau métallique à double tranchant avec un bord en dent de scie, traversant de part en part le tiers moyen de l´avant-bras dans une direction oblique de la face médiale à la face antérolatérale. On ne notait pas de lésions osseuses (Figure 2).

**Figure 3:**
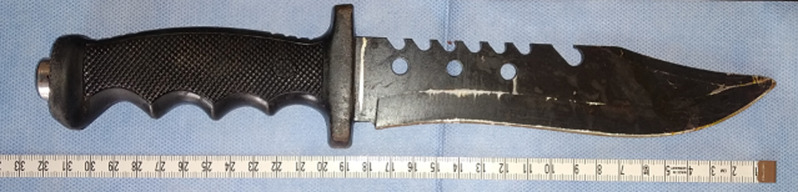
l’objet traumatisant après ablation au bloc opératoire

**Intervention thérapeutique et suivi:** des antalgiques et la prophylaxie antitétanique avaient été administrées au patient. L´exploration chirurgicale avait été faite. Nous avions réalisé un élargissement et une dissection dans le sens longitudinal des plaies, une protection des tissus par des compresses et retrait du couteau sans résistance. La lame du couteau mesurait 19/4 cm (Figure 3). Comme lésions internes, on notait: une section partielle avec contusion des corps musculaires du rond pronateur, du supinateur, des fléchisseurs superficiels et profonds des doigts. Une section totale du corps musculaire du fléchisseur ulnaire du carpe. Une section de l´artère radiale et la branche superficielle du nerf radial. Le paquet vasculo-nerveux ulnaire et le nerf médian ont été repérés et ne présentaient pas de lésions.

**Figure 4:**
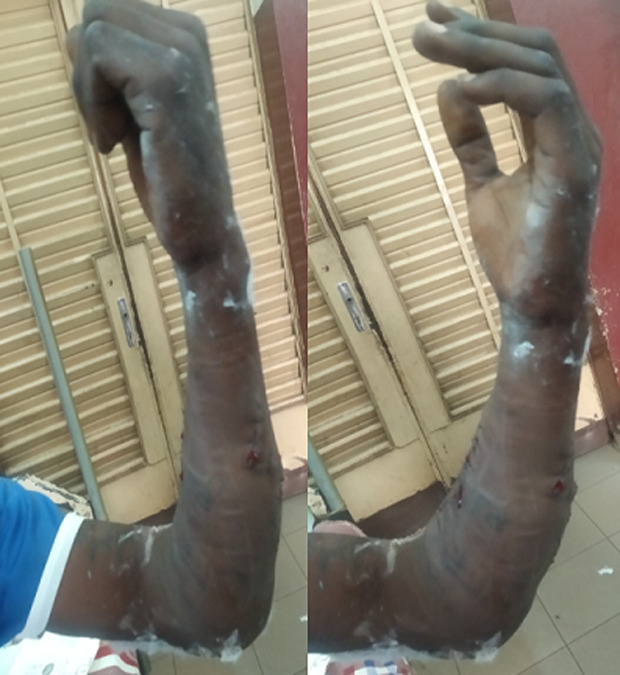
état postopératoire de l’avant-bras avec rétablissement de la flexion et l’extension des doigts

Après parage, la réparation vasculaire a été faite par une suture termino-terminale au fil nylon 5/0 avec un test de Patency positif. La réparation nerveuse faite par une suture épineurale au fil nylon 5/0. La suture musculaire faite par des points en X au fil résorbable 1. Une fasciotomie sous cutanée a été réalisée avant la fermeture cutanée par des points séparés, avec mise en place d´une attelle plâtrée postérieure en brachio-palmaire, poignet en flexion de 30 degrés. Une antibioprophylaxie peropératoire avec de l´amoxicilline-acide clavulanique et du métronidazole a été administrée. Un protocole de rééducation protégé selon Duran a débuté en postopératoire immédiat, poursuivi jusqu'à 6 semaines avec ablation de l´attelle à 3 semaines (Figure 4).

**Consentement du patient:** il a été obtenu après explication et garantie de l´anonymat.

## Discussion

Les agressions physiques avec usage d´armes blanches représentent près de la moitié des cas de coups et blessures dans notre contexte [2]. Il s´agit souvent des jeunes hommes actifs et près de la moitié des plaies pénétrantes concerne les extrémités [3]. Chez notre patient il s´agissait d´une rixe avec coup de couteau resté planté dans son avant-bras. Ce segment du membre supérieur est souvent blessé, il est plus exposé dans le mécanisme reflexe de protection de la tête par élévation et pronation de l´avant-bras. L´examen physique au service des urgences de ces traumatismes peut être difficile à cause de la douleur et l´état émotionnel du patient, pouvant entrainer une mésestimation des lésions neurologiques [4]. Les atteintes nerveuses et vasculaires ne sont pas rares dans les traumatismes pénétrants de l´avant-bras [3, 5]. La présence de signes physiques patents de lésions vasculaires est une des indications d´exploration chirurgicale en urgence [6].

L´abolition du pouls radial dans notre cas était le signe palpatoire retrouvé, nous n´avions pas noté d´hématome pulsatile ou expansif. Les examens paracliniques viennent compléter l´examen physique en précisant le type, la taille et le siège de l´objet traumatisant, un angioscanner peut préciser le siège de la lésion vasculaire [1, 6]. Dans notre cas, la radiographie a révélé l´absence de lésions osseuses, et à préciser les caractéristiques du couteau; à double tranchants avec une lame crantée. Ce type de couteau pose une difficulté supplémentaire lors de l´ablation, le risque de survenue de lésions iatrogènes des parties molles est élevé, compte tenu des multiples aspérités pointues et coupantes présentes.

Notre exploration chirurgicale avait retrouvée des sections des corps musculaires, la lame du couteau passant ainsi en arrière du paquet vasculo-nerveux ulnaire et du nerf médian. Sur ce segment de membre, une lésion vasculaire doit faire suspecter une lésion nerveuse satellite [4], ce qui est le cas dans notre observation. La lésion vasculaire retrouvée était la section de l´artère radiale à 5 cm en dessous du pli du coude. La lésion nerveuse concernait la branche superficielle sensitive du nerf radial sur la même projection de la lésion vasculaire radiale. Un travail fait sur les traumatismes pénétrants du membre supérieur montrait de lésions artérielles sur l´avant-bras dans 21,2% [3]. Iconomou *et al*. dans une étude sur les traumatismes de l´avant-bras par des objets tranchants retrouvait une lésion du nerf radial dans 25% et des fléchisseurs ulnaire du carpe, superficiels et profonds des doigts dans 57% des cas [7]. Certains auteurs tels que Gürbüz *et al*. avaient opté pour un retrait du corps étranger au bloc et une exploration chirurgicale même sans signes cliniques de lésions neurovasculaires [8]. Notre observation montre la dangerosité et le caractère multilésionnel des traumatismes transfixiants de l´avant-bras par des couteaux et le risque iatrogène élevé au cours de l´ablation. La prise en charge pouvant ainsi faire appel à des réparations microchirurgicales [9], garantissant une meilleure récupération fonctionnelle du membre traumatisé avec une rééducation bien menée.

## Conclusion

Les plaies transfixiantes de l´avant-bras par objet tranchant implanté ou non, ne doivent pas être négligées. Un examen clinique minutieux, complétée par l´imagerie permettent de diagnostiquer des lésions musculaires et neurovasculaires souvent graves. L´ablation au bloc opératoire du corps étranger est recommandée, afin de réduire le risque de lésions iatrogènes et de traiter au mieux les lésions présentes.
